# Stressors and resources related to academic studies and improvements suggested by medical students: a qualitative study

**DOI:** 10.1186/s12909-019-1747-z

**Published:** 2019-08-20

**Authors:** Jeannette Weber, Stefanie Skodda, Thomas Muth, Peter Angerer, Adrian Loerbroks

**Affiliations:** 1 Institute of Occupational, Social and Environmental Medicine, Centre for Health and Society, Faculty of Medicine, Heinrich-Heine-University of Düsseldorf, Moorenstraße 5, 40225 Düsseldorf, Germany; 2 Mannheim Institute of Public Health, Social and Preventive Medicine, Mannheim Medical Faculty, Heidelberg University, Mannheim, Germany

**Keywords:** Qualitative research, Focus groups, Stress, psychological, Health resources, Education, medical

## Abstract

**Background:**

Prior evidence suggests that medical students’ mental health is poor and deteriorates during the course of academic studies. This qualitative study therefore aims to improve our understanding of medical students’ perceptions of i) stressors related to their academic studies, ii) resources that may facilitate coping with those stressors and iii) suggestions to potentially reduce stress.

**Methods:**

Eight focus groups were conducted with medical students enrolled at a medical school in Germany until thematic saturation was reached. A topic guide was used to facilitate the discussion. Subsequently, focus group discussions were transcribed and content-analyzed using MaxQDA.

**Results:**

Organizational factors especially related to inadequate information flow as well as exams (e.g. repeat exams, scheduling, perceived unfair grading), poor theoretical and practical teaching quality, time and performance pressure, social interactions and individual characteristics (e.g. self-expectations, fear of failure) emerged as major contributors to stress. Resources perceived to facilitate coping with those stressors pertained to some other organizational aspects (e.g. flexibility, availability of contact persons), career prospects, practical training, social support, personal characteristics (e.g. knowledge base, past experience) and leisure time. Suggestions for improvement related primarily to organizational measures rather than individual-level measures.

**Conclusions:**

Besides well-known stressors (e.g. exams and high performance pressure), some new aspects emerged from our study including stress related to organizational factors and repeat exams. Accordingly, students’ wishes for organizational-level interventions, including better information systems and better interweaving of practical and theoretical education, could be first target areas for improvement.

**Electronic supplementary material:**

The online version of this article (10.1186/s12909-019-1747-z) contains supplementary material, which is available to authorized users.

## Background

Medical studies are perceived as very stressful, e.g., due to the associated high workload, emotional demands and exposure to death and ill-health [1]. It is therefore not surprising that previous research has observed a decline of mental health in medical students throughout the course of their academic studies, finally reaching levels which are lower than among the general population of similar age [1–4]. In a significant number of studies, distress and poor mental well-being were further associated with suicidal ideation and exit from medical schools [5–7]. Furthermore, concerns regarding implications for adequate patient care during and even after practical medical training were raised [8, 9]. Therefore, interventions to improve study conditions and strengthen students’ abilities to cope with them are urgently needed.

In quantitative research (e.g. surveys), academic factors were identified as main stressors for medical students including exams, time management, a high workload, dissatisfaction with lectures as well as selection and performance pressure [10–13]. Psychosocial resources are factors that have an intrinsic value or are useful to pursue goals and cope with demands and stress in everyday life [14]. They may include self-esteem, health, skills, knowledge, social support or other factors and may decrease students’ stress. During medical education, resources such as joy, optimism, social support and self-care (e.g. adequate nutrition, physical activity, social relationships) have been found to be associated with reduced perceived stress and to buffer against potentially negative effects of stress on mental health [4, 15, 16]. Further, several intervention studies have demonstrated the effectiveness of mindfulness-based training to reduce stress in medical students [17].

Those quantitative studies have provided important data that helps to identify stressors related to medical studies (i.e. observational studies) and to test approaches to reduce students’ distress (i.e. experimental studies). However, quantitative studies usually rely on pre-conceived notions regarding relevant stressors or resources. Therefore, stressors, resources and starting points for interventions which may be specifically important for medical students themselves might have been overlooked by using standardized data collection tools [18]. Starting points for interventions and health promotion may include the enhancement of resources that are perceived to be useful by medical students themselves. Furthermore, only interventions that are considered useful by medical students will be accepted and utilized by members of this population. Qualitative research, by contrast, offers the opportunity to gain such in-depth information without prior pre-conceived notions regarding stressors, resources and intervention needs due to its focus on the experience of individuals in everyday life [18, 19]. In particular focus groups constitute a highly exploratory approach and are well suited to study common experiences [20]. Focus groups are likely highly effective in investigating potential strategies for intervention because a broad consensus can emerge from participants discussing and reflecting upon each other’s suggestions and wishes.

So far, several qualitative studies have addressed specific stressors during practical as well as theoretical medical education in Europe. Using individual in-depth interviews, two studies have revealed an overload of routine activities, lack of skills and knowledge to perform certain tasks, role conflicts, the feeling of having to prove oneself, and loneliness as specific stressors during practical training [21, 22]. Two further studies have also examined theoretical medical education and suggested further stressors including a high workload due to studying for exams and acquisition of new knowledge and skills, transition from school to university as well as regulations about absence [23, 24]. Also several types of psychosocial resources and suggestions for improvement regarding the practical year have been identified [22]. However and to the best of our knowledge, only one study has explored suggestions for improvement and psychosocial resources to cope with stress during earlier parts of medical education [24]. In that study, data collection included only two focus groups with one of them lacking a complete transcript [24]. Therefore, data saturation is questionable (i.e. the point when no more information is expected by conducting further data collection), which represents a quality criterion of qualitative research [25]. We therefore aim to expand those preliminary findings based on a more in-depth inquiry building on data collection through focus groups until data saturation is reasonably achieved. Doing so, we will explore medical students’ perceptions of i) stressors related to their academic studies, ii) resources that help to handle those stressors and iii) suggestions for improvements that may potentially reduce the stress experienced during medical studies.

## Methods

### Study participants

Study participants were recruited from the Medical School at the University of Düsseldorf in Germany. Each year, approximately 400 students embark on their studies at our medical school. In 2013, a new competence-oriented curriculum was introduced. That new curriculum builds on an interdisciplinary approach and emphasizes hands-on training (i.e. earlier in the curriculum and to a larger extent). Furthermore, internships with a total duration of four months in hospitals, family practices and outpatient care are mandatory in all German medical schools. Moreover, before graduation a full practical year in medical care has to be completed. Study participants were approached via social media or recruited through personal contacts of doctoral students (Stefanie Skodda (SS), Christin Bergmann (CB; see acknowledgements)) of the research team. Regretfully, we do not know how many students were exposed to the recruitment materials and therefore participation rates cannot be calculated. In order to facilitate engagement of all study participants in the discussions, each focus group was comprised of participants who studied in the same academic year, due to pre-existing familiarity with each other and their common experiences. Six to eleven students participated in each focus group, except for one small focus group with only two participants. Inclusion criterion was enrollment in human medical studies. No further selection criteria were applied. Participants were compensated for their time with a cinema or bookshop voucher.

In total, eight focus groups were conducted with 68 participants. Four focus groups were conducted with students in the fifth year, two with students in the second year and two with students in the last month of the first academic year of education. We also gathered additional information regarding age and sex from participants of the last six of our eight focus groups (respectively three focus groups with students in the fifth year, one with students in the second year and two with student in the first year of medical education). The mean age of those participants was 24 years (range 18–34 years) and 77% were female and 23% were male. The first two focus groups were conducted following exactly the methods as described below, but with the aim to simply learn about students’ experiences in their medical studies. For this reason, sociodemographic data was not gathered from the first two focus groups.

### Study design

Focus groups were conducted between November 2013 and July 2015 until data saturation was reached. Prior to the study, a topic guide was developed by Adrian Loerbroks (AL) and Thomas Muth (TM) and two medical students (SS and CB) to facilitate focus group discussions (the topic guide can be found in Additional file 1). AL and TM have a vast knowledge of the current stress literature, are experienced in qualitative research [26, 27], and are teaching staff of the medical school. Furthermore, TM has close contact with medical students in his role as coordinator and contact person for elective courses at our institute. We can further assume that most study participants were aware that TM’s main research focus pertains to medical students’ health. Focus groups were held at a conference room at our institute and were facilitated by TM while SS and CB took field notes. Focus groups were conducted and content-analyzed in German. Relevant quotes were translated into English by a certified translator after data analysis (see acknowledgements). An open introductory question was used to initiate discussions amongst study participants, asking them to reflect on their studies, how they had experienced them so far, what had been difficult and what had helped them to cope with their studies. All participants were encouraged to contribute to discussions early. Whenever appropriate, the facilitator explored a topic in greater depth during the course of each focus group to direct the discussion to underlying causes of perceived stress, resources used to cope with this stress, balance between private and academic life^1^ and finally to suggestions for improvement. Each focus group lasted for about 90 min.

### Data analysis

All focus groups were digitally recorded and transcribed. Subsequently, the material was analyzed by Jeannette Weber (JW) following established approaches to qualitative content analysis [28, 29] using the MaxQDA 12 software package. JW has an educational background in public health and experience in occupational health research. In a first step, our research questions as specified in the topic guide were included as main categories (i.e. deductive coding): “stressors”, “resources” and “suggestions for improvement”. During analysis, those categories were further broken down into sub-categories by inductive category formation. After coding of the first four focus groups was completed, those sub-categories were revised and when appropriate corresponding text passages were reanalyzed. Then, the final four focus groups were analyzed and, if needed, additional sub-categories were created. Subsequently, the coding scheme was reviewed by AL and some further adaptations were discussed. Finally, a second coding round was conducted by JW. However, this second coding round entailed only few adaptations. Therefore, two coding rounds were considered to be sufficient. Corrections and feedback on transcripts and research findings were not obtained from study participants due to logistic constraints.

The completed checklist of consolidated criteria for reporting qualitative research (COREQ; [30]) can be found in Additional file 2.

## Results

**Fig. 1:**
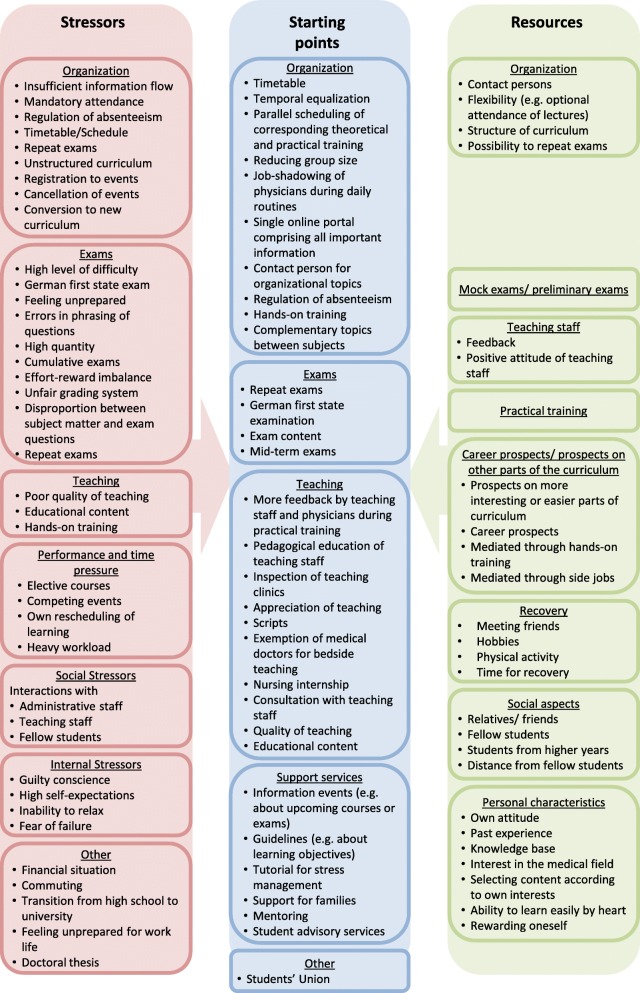
Coding system with main- and sub-categories. Only sub-categories at first and second level are shown

The coding system including clustering of main- and sub-categories at first and second level can be found in Fig. 1. Additional quotes that are not cited in the text can be found in Additional file 3.

### Stressors

#### Organizational stressors

Students perceived an insufficient information flow from administration and teaching staff which was characterized by missing information, inadequate feedback of results, delayed provision and updating of schedules, a lack of contact persons and conflicting information depending on which staff were asked. Furthermore, students criticized that there was no single standardized source of information, but reported that information about courses and examinations is spread across various online portals and web pages. Furthermore, some students felt stressed by the varying types of teaching materials (e.g. books, scripts, PowerPoint presentations) and lack of information regarding which of those materials ought to be used to prepare best for exams. This insufficient information flow resulted in rumors and hearsay, uncertainty of what is expected and fear of missing out on important information. Furthermore, mandatory attendance and regulation of absenteeism were perceived as burdensome.

*“I think […]*, *that it’s always somewhat unclear what’s going on. One asks about something and gets 10,000 different answers. Everyone says something else, and when you google it or check it on the website you don’t find anything. For example, not being able to attend, there are different regulations for each subject. How many times are you allowed to be absent. OK, 15% is allowed in general, but there are subjects where they’re calculating 15% per module. Then there are subjects where they’re calculating 15% for all 12 modules. In this case one can check for oneself, how often do I have to attend here and there and how many times can I be absent. Of course, you don’t know exactly, because we don’t have the schedules for all 12 modules yet […]*. *” (Focus group (FG) 4 - Study year (Y) 1).*

The timetable was perceived as an additional stressor due to a large number of teaching events in short periods of time or even simultaneous events.

*“Well my group had a module with an internship in a practice and my impression was that I miss out on a lot of things during that week. I was in the practice every day until 7 p.m. and […* *]* *I somehow felt that I don’t have enough time to work through everything during that week. I also had to do a sonography exam during that week. I think it’s totally crazy to organize it in a way that it’s scheduled for the middle of the semester.” (FG 5-Y2).*

Study participants also highlighted issues regarding repeat exams, which were usually scheduled at inconvenient times during the academic year. Some students criticized that the schedule changes on a weekly basis. Particularly those students who commute between university and their home town felt stressed when only one event was scheduled on a day or when unnecessary and long breaks were scheduled between different events (Quote (Q) 1, see Additional file 3). Furthermore, students believed that subjects were unstructured in terms of content and time and perceived a gap between theory and practice.

*“One thing that continues to annoy me […* *]* *is the separation of practice from theory within the modules. That they are not connected at all is something that I find irritating again and again, […* *]* *Then you have a module about the head and afterwards you practice in, I don’t know, nephrology, radiotherapy, orthopaedics, and psychiatry, yes psychiatry would also be useful. But that there are no connections, I always find that annoying, also because then you’re often in a situation where you have to say, sorry, I haven’t learned that yet, I can’t say anything about it. Personally, I always find this very inconvenient and it doesn’t help me at all for getting in-depth knowledge on something I studied for three weeks.” (FG2-Y5).*

In addition, registration to events and cancellations of events, especially when students were not informed beforehand, were mentioned as further stressors. Some students believed that those organizational deficits were associated with the conversion of the standard curriculum to a new curriculum.

#### Exams

Difficult exams, the German first state examination (“Physikum”), feelings of being unprepared, errors in the phrasing of exam questions as well as a high quantity of exams emerged as further stressors. In addition, cumulative exams (i.e., a number of exams that add up to a final grade) seemed to increase pressure because students had to wait until the end of the study block to know whether they passed the exam (Q2). The feeling of an imbalance between one’s efforts and rewards was often expressed as large amounts of time spent learning did not necessarily correspond with equivalent grades or passing exams.

*“Before each final exam of a module you’ll go to your limit. I have never attended a module without someone crying because they had a nervous breakdown. It becomes, I don’t know, quite emotional. […]* *And compared to this I think the results aren’t great either. I mean people go to their limits and still there are so many who fail.” (FG4-Y1).*

Furthermore, unfair grading systems - i.e. the grade being partly contingent upon the specific examiner - were perceived to increase stress.

*“I mean, I have already begun to wonder who will be in charge of my oral equivalency exam next year. Because no one can tell me that there are equal opportunities. Well, it makes a big difference who sits in front of you and asks questions, and these are things that create more stress than the preparation itself.” (FG5-Y2).*

A disproportion between subject matter and number of exam questions was also alluded to. Furthermore, study participants felt highly stressed due to repeat exams because a) they were scheduled at inconvenient times, b) one has to learn for them again simultaneously with other regular exams, which increases time pressure (Q3), and c) of the option to repeat exams only twice. After failing the same exam for the third time, students are excluded from medical studies at any medical school in Germany.

*“This is simply the pressure one feels, that you know that if you fail three times, something that happens really, really quickly, then you will never be able to do it again. If I failed three times, I have no idea what I would do then.” (FG8-Y2).*

#### Teaching

Poor quality of teaching, including lectures, lecture slides and scripts, as well as lack of guidance and supervision was often perceived as demotivating (Q4). Furthermore, study participants felt that they were not well prepared for exams (Q5). Some educational content was experienced to be inadequate. Specifically, according to participants’ views, unimportant and easy topics are frequently and strongly emphasized whereas important and more complex issues remain unaddressed.

*“Yes, and honestly, it’s unbelievably discouraging when you’re told that you don’t really need this for practicing, then I ask myself, why don’t we learn the things that are really important for practicing.” (FG6-Y1).*

Due to the perceived frequent emphasis on supposedly unimportant topics a feeling of time waste was often mentioned, especially in association with mandatory attendance. Furthermore, some students expressed that they are bored and not intellectually challenged due to the requirement of learning a lot of content simply by heart (Q6). Many participants pointed to poor supervision during practical tasks.

*“Well, I find it annoying that we have no supervision and that we have to take care of everything by ourselves, you have to realize, it’s not always their fault, perhaps they would like to help but they just don’t have enough time. That’s annoying.” (FG1-Y5).*

Some students also expressed that some physicians are unfriendly, demotivating and that they give them a feeling of being bothersome during their practical tasks.

*“When you’re looking for a physician for three weeks who just makes you feel that you’re a burden (,) I do find this extremely discouraging.” (FG1-Y5).*

Furthermore, some students felt that the mandatory internship at the general practitioner is ineffective. They complained about a lack of responsibility and the inability to adequately perform their tasks for university. If they were allowed to perform some medical examinations, they felt uncomfortable due to a lack of experience and permission to discuss their diagnoses with the patient.

*“Sometimes it was really inconvenient to go to a patient and say, OK, I’m a student and I will examine you. And then they ask me, what do you think. Well, I am not allowed to express an opinion. What. No. Well I sometimes found this very inconvenient to sit there with my partial knowledge and to pretend that I could do a perfect examination, but I am not allowed to diagnose them.” (FG5-Y2).*

In addition, the nursing internship of three months was experienced as time-consuming and some students reported that responsibilities during the internship were not clearly communicated beforehand.

*“For example, in my case I divided the [nursing] internship into three parts and in one case they wanted me to work thirty days in a row for eight hours per day without a break. Not even two days off in between, although as I know now I would have had a right to them. I basically had no idea what I was allowed to do and what I wasn’t allowed to do. In the end […* *]* *nurses sometimes forced me to do things I wasn’t allowed to do, e.g. giving injections to prevent thrombosis.” (FG8-Y2).*

#### Time and performance pressure

The feeling of time pressure caused by competing events, own rescheduling of learning, elective courses and a heavy workload due to a high amount of subjects as well as very comprehensive single subjects was often expressed (Q7). This seemed to result in a lack of time for private life and to learn guided by one’s own interests (Q8), in a lack of motivation (Q9), and in learning on short-term memory.

*“I think here we really bring ‘bulimic studying’ to perfection. During the three weeks before the written exam I stuff myself with everything. Study all night. I try to find time for everything. I can’t tell you anything anymore about locomotor system or TB1 or TB3. I can’t tell you what I learned last week because I’ve already forgotten it. We try to learn so much in such a short period of time. And we have to concentrate on a new subject immediately afterwards. That there’s no chance to remember what you learned in the last modules or to enhance it.” (FG4-Y1).*

Many students also alluded to a lack of recreational time due to courses, internships and exams during non-lecture periods and emphasized that they feel exhausted at the end of the academic term. Furthermore, they expressed the feeling of performance pressure, which occurred due to the scheduling of repeat exams and simultaneous scheduling of lecture periods, exams and internships. They further expressed the thought that this performance pressure is deliberately built up by the medical school to test students’ stamina and to thereby initiate selection according to stamina.

*“The impression is that there’s pressure to simply select and I really find this (,) to be quite honest I have come to find this a bit annoying.” (FG8-Y2).*

#### Social stressors

Interactions with administrative staff were heavily criticized and unfriendliness as well as a lack of appreciation, respect and support was experienced.

*“Well you’re not really appreciated here. They rush you through it and, uh, when you don’t function you’ll be punished.” (FG8-Y2).*

Additionally, a bad atmosphere between teaching staff and between students and teaching staff was reported (Q10). Furthermore, fellow students were identified as additional stressors. First, stress seemed to develop due to one’s own comparison with fellow students in terms of time spent for learning and learning progress.

*“In the week before the exam I have at least one nervous breakdown per day, because I sit there and start crying, because when I talk to friends who are more advanced I see that there are so many things I still have to do. You have no idea what they’re talking about […]*” *(FG4-Y1).*

Second, most social contacts involve other medical students and therefore conversations always addressed medical studies even in private life.

#### Internal stressors

Internal stressors seemed to involve a guilty conscience when taking a break from learning due to high performance pressure, high self-expectations regarding one’s performance during medical studies (Q11), inability to relax and fear of failure regarding exams (Q12) and as a physician in the future.

*“You always think about it, OK, you didn’t study for two hours. Now you have a bad conscience and having a bad conscience when you spent ten hours studying and did something else for two hours, somehow that’s sick.” (FG7-Y5).*

*“When I meet with friends or do something with my family, then I always think in the back of my mind that I could just as well study. You can’t enjoy it.” (FG6-Y1).*

#### Additional stressors

Finally some additional stressors were highlighted including the financial situation (Q13), commuting between university and one’s home town, transition from high school to university, writing a doctoral thesis (Q14), which in Germany is often performed during medical studies, and feeling unprepared for future work life.

*“Somehow, I think that (,) for me it’s really sad, because after a very short time I will be a physician and I think OK, concerning the eyes, for example, there’s a knowledge gap. I find it very embarrassing because if you ask me about it or I have to say something about it, then I don’t know anything about it and because of all the organizational stuff this went wrong a bit.” (FG3-Y5).*

### Resources

#### Organizational aspects

A well-structured curriculum, combining practical and theoretical training and having a block by block structure, was appreciated by study participants. They argued that this helped them to deepen their knowledge, to really concentrate on one subject and to feel certain on what to expect at exams.

*“What you said is quite right, these eight-week modules, I found it so relaxing to study there […]*. *That’s great. That’s so nice because it’s a combination of both. There are good modules for practicing, because they do a great job organizing, and you have theory, but it’s not too much and you can repeat in between.” (FG3-Y5).*

Flexibility regarding optional attendance of lectures (Q15), the possibility of repeat exams (Q16) and having a contact person for each subject were named as additional organizational resources.

*“Yes. Basically, I have to say that I found it really great that in the beginning professor X stood there and said ‘I am in charge of this module and if you have any questions please contact me’. In the other modules, it was not clear (other participants: agreement – yes) who you could contact. But basically, you simply have someone of whom you knew you could contact them. And I must say this module was the first one where this happened. I found this extremely helpful.” (FG5-Y2).*

#### Practical training and career prospects

Practical training and internships were viewed as opportunities to practice what has been learnt. It seemed to help to remember and to better understand content covered in teaching. Furthermore, practical training, internships and side jobs seemed to motivate study participants to proceed further with their education because it reinforced their career choice (Q17). Good career prospects in the medical field were also repeatedly named as important motivation to proceed further with studies despite increased stress and workload.

*“I believe I simply know that it’s the right thing and that you look forward to it, and I think being a physician is stressful and perhaps frustrating and all kinds of things, but it’s a great job. I really look forward to it because it’s not a job that involves moving piles of pallets from A to B, or checking if some part for cars arrived, it’s a job where I will rush home at night and I will say you did not change the world but you made a certain difference for a patient and I think this is (,) this will be a great feeling. And we have this with small things, during part-time jobs all of us (,) or in a practical module, or during a clinical elective or something like this, but it’s very satisfying and this is what helps you to get through with your studies.” (FG2-Y5).*

In addition, prospects on easier and more interesting parts of the curriculum seemed to help some participants to carry on (Q18).

#### Social aspects

Contact with relatives and friends seemed to help study participants to cope with increased stress due to their studies (Q19). Additionally, also social interactions with students from higher years and fellow students were named as important resources. They assured social contact and exchange of important information (Q20–21). Furthermore, students felt less lonely and were able to share their stress, anger and fears regarding medical studies with like-minded persons.

*“What really helped me is that I wasn’t the only one who suffered. I always think that sharing suffering, that’s what (,) while studying medicine it becomes very obvious that it’s extremely helpful when others are in the same boat and also rant.” (FG3-Y5).*

Conversely, some participants believed that distance from fellow students helped them to cope with and get away from stress due to medical studies.

*“But it was the most important thing, I completely isolated myself from my fellow students, I really didn’t want to meet them because I knew they are also stressed out by studying and they are all afraid and I didn’t want to catch their panic. And this really worked out.” (FG7-Y5).*

#### Personal characteristics

A relaxed attitude involving lower self-expectations, acceptance when things are not going so well (Q22) and less comparison with other students was believed to decrease stress. Also past experiences seemed to help students cope with high demands related to medical studies (Q23). Furthermore, a good knowledge base, interest in the medical field, partial control of learning content according to one’s own interests, ability to learn easily by heart and rewarding oneself were named as further resources.

#### Recovery

Hobbies, physical activity, meeting friends, holidays, recreational time and breaks during learning phases were thought to be important by the majority of study participants.

#### Additional resources

Committed teaching staff and medical doctors who mentor during practical training seem to motivate study participants to carry on and help them with learning content (Q24).

*“I always find when I meet a physician who is very motivated and who is able to explain things very well, then I really enjoy it and I think wow, that’s a great kind of studies. And I look forward to becoming a physician one day.” (FG7-Y5).*

Personal feedback from teaching staff was also regarded to be helpful. Furthermore, preliminary tests (Q25) and mock exams were regarded as opportunities to practice and to prepare for major exams.

*“[…* *]* *uploaded an exam for preparation, twenty questions were assigned by chance and you could answer them, and finally you could see the right and wrong answers and who failed. I think that prior to the locomotor system exam I did this 38 times. I felt so incredibly safe, I was not afraid of the exam at all.” (FG6-Y1).*

### Suggestions for improvement

The study participants made a broad range and to some extent also very specific suggestions for improvement. The reproduction of all suggestions would by far exceed the scope of this article and thus the presentation is limited to the most salient findings.

#### Organizational factors

Regarding timetables, study participants wished for earlier announcement of examination dates, more flexibility (Q26) and less mandatory events (Q27). In addition, temporal equalization in placing subjects from time-consuming terms into more relaxed terms and increasing lecture periods and study blocks was suggested (Q28). However, some participants also wished that no further events should be scheduled for non-lecture periods (Q29). Furthermore, parallel scheduling of corresponding theoretical and practical training was requested. A group size of less than 15 students during bedside teaching was regarded to increase opportunities for learning (Q30). Furthermore, some participants wished to be taken on daily work routines of medical doctors and to have more guidance during practical tasks (Q31). They further wished that the teaching staff of different subjects would discuss and coordinate their content to ensure that topics between subjects are complementary and non-overlapping. Furthermore, to improve information flow students suggested that there should be i) one single online portal which comprises all important information (Q32) and ii) a single designated contact person for organizational issues.

#### Exams

Several suggestions were expressed regarding repeat exams including a larger number of attempts, prompt scheduling after the first exam and the possibility to unsubscribe from exams. Furthermore, a wish was stated that exams should assess understanding and more relevant topics rather than rote recall. Further suggestions included a smaller number or less importance of mid-term exams (Q33).

#### Teaching

To increase the quality of teaching, better pedagogical education of teaching staff, inspection of teaching clinics, higher appreciation of teaching and exemption of medical doctors from day-to-day business for bedside teaching was proposed. Students also wished that their evaluations of study courses are incorporated in continuous development of the curriculum. Some students suggested to reduce the nursing internship to one month and to inform nurses about learning objectives and range of authority to decrease strain on medical students. Furthermore, some students wished for more consultation with teaching staff including opportunities to pose questions after lectures. In addition, suggestions pertaining to more basic teaching (Q34), soft skills and medical skills (e.g. thoracic drainage or sonography, Q35) as well as tutorials and practical training to deepen and discuss learning content were made.

*“Could it perhaps be useful if there were regular seminars for the different subjects, by simply (.) […* *]* *I think oral presentations don’t make sense because no one listens, but mid-term tests, one prepares for them, one deals with them, then you do the test. Then we could simply discuss them afterwards or exchange ideas or talk about them, so that it’s sustainable. Step by step. Building in-depth knowledge by repetition throughout the curriculum. […* *]* *To be quite honest, when you discuss a subject with other people, then it consolidates.” (FG4-Y1).*

#### Support services

Some students asked for information events, especially in the beginning of the academic term, to increase information flow about inter alia upcoming subjects, exams or elective courses.

*“Just a simple suggestion (,) if one could simply say at the beginning of the semester perhaps the dean will attend in this case, will spend two hours of time and come to the lecture hall. He will give an introductory lecture, just like at the beginning of the first semester, but that we simply are told this semester encompasses these subjects, these are the dates of the written exams. Then you also have the chance to ask questions if anything is unclear.” (FG8-Y2).*

Furthermore, students wished for guidelines about upcoming subjects and learning objectives, a tutorial for stress management, support for families, mentoring and student advisory services (Q36).

*“OK, well, yes, that you receive a kind of schedule in the beginning, after each semester this is what you should be able to tick off and this is what you should have done.*” *(FG3-Y5).*

*“What I think another issue is, I don’t know if this could be put into practice because of the large number of students. It would be great if every student could have a physician as a mentor […* *]* *That would be awesome, then you would have access. Then you would have a person to provide stability and who says, this and that is what you need to know. Someone who looks over your shoulder. Who, I don’t know. Then it would be easier for us to transition between theory and practice. We could better fulfil the expectations.” (FG7-Y5).*

## Discussion

A large number and large variety of stressors, resources and resultant suggestions for improvement were identified based on eight focus groups. Most stressors involved organizational structures. Furthermore, exams, poor quality of teaching and lack of guidance during practical training, time and performance pressure due to a high workload, social stressors due to interactions with administration, teaching staff and fellow students and some internal stressors (e.g. high self-expectations, fear of failure, inability to relax) emerged. In contrast, organizational aspects such as flexibility and a combination of theoretical and practical training were perceived as important resources. Additionally, career prospects often conveyed by internships or side jobs, social support by family members, friends and fellow students, personal characteristics and recovery through hobbies, physical activity and meeting friends were perceived as helpful to cope with stress. Accordingly, suggestions for improvement often involved organizational aspects, exams and teaching quality, but also included requests for support services.

Our results are in line with previous qualitative studies on this research topic [23, 24, 31, 32], which implies that identified stressors and resources generally apply to students at various medical schools in and outside of Germany. Furthermore, some new themes emerged related to organizational structures especially regarding information flow, repeat exams, perceived effort-reward imbalance and challenges due to commuting. The fact that we found a larger scope of themes may indicate that a particularly thorough thematic saturation was achieved based on our eight focus groups. Alternatively, one may speculate that additional themes are of contextual nature and thus specific for the medical school where this study was carried out. For example, daily commuting between one’s home town and the university is very common in the area where our medical school is located (i.e. the Rhine-Ruhr area in Germany) and may be less of a salient issue in other university cities outside this area. Furthermore, some of our participants were in the first year of a new study curriculum at this particular university. Therefore and as acknowledged by some study participants, challenges and stress related to organizational structures may have partly been caused by this recent re-design of the curriculum.

Not surprisingly, exams have often been identified as major stressors in higher education and our study is not an exception [23, 24, 31, 33]. However, not only the high quantity and difficulty of exams were perceived as stressful, but we also identified some new aspects on how exams contribute to stress, in particular among medical students. Those new aspects were mainly associated with organizational factors that pertain to repeat exams and grading systems that were perceived to be unfair. On the one hand, the option to repeat failed exams was referred to as a resource by several students. On the other hand, the fact that only three attempts are granted was heavily criticized. This issue may have been aggravated by the fact that students were not able to decide by themselves on when to repeat exams, but were automatically registered if they had not passed the first exam. Furthermore, medical certificates were needed when students did not feel well enough to participate in exams. To reduce this pressure, it was recently decided by this particular medical school that students should register for exams by themselves. Furthermore, supposedly unfair grading and an imbalance between time and effort that was put into the preparation of exams and subsequent results were highly criticized. This corresponds to the effort-reward imbalance (ERI) model, a well-established stress model in occupational contexts, which assumes that the combination of high effort and low reward results in emotional distress [34]. The ERI model has recently been adapted to academic settings and has shown its utility to capture stress in medical students [35].

Additionally, the emphasis on poor teaching quality is worrisome. In accordance with another qualitative study with medical students in Germany, students criticized a strong focus on topics that are perceived to be unimportant and asked for prioritization on basic knowledge, skills and clinical relevance [24]. However, it may be questionable whether students are able to fully grasp the relevance of specific content and a first step may include better communication of their practical relevance. Introduction of re-designed curricula represents an approach taken by an increasing number of German universities to exert a stronger focus on practical training and to combine preclinical and clinical education. Practical training seems to motivate students and has been shown to promote clinical thinking and learning [36]. In this context side jobs and internships were alluded to as important resources during our focus groups. However, it seems that the implementation of the new curriculum at this particular university has been challenging and stressful to students. Students still wished that topics between subjects and the scheduling of courses are better matched to effectively combine preclinical and clinical education as well as theoretical education with corresponding tutorials and practical courses. Furthermore, a perceived lack of support and guidance during practical training has been expressed and has partially been attributed to contextual problems involving the shortage of health care professionals in Germany.

Clinical settings involve strong hierarchies and especially physicians in lower positions frequently report exposure to workplace bullying [37]. The mistreatment of medical students is not a new problem and is accompanied by a widespread attitude among the medical professional society that a rough educational climate is an effective and acceptable teaching strategy [38, 39]. Our study expands the current knowledge by highlighting that this unfavorable atmosphere is already perceived to be established during undergraduate medical education through unfair treatment, allegedly unfair grading, inconvenient scheduling of exams, unclear regulations regarding absenteeism and a lack of support. Furthermore, performance pressure has also been identified as a major stressor in previous quantitative research [11]. Such selection pressure was also experienced by our study participants, who felt that a high workload was utilized to test students’ stamina and to select students based on their stamina. By contrast, committed and supportive teaching staff and physicians were mentioned as a resource to cope with medical studies.

Students expressed that those stressors had several sequelae including lack of motivation, distress, fear, exhaustion, learning and recalling content only for the short-term, inability to relax after high effort and feeling unprepared for examinations and working life. This is alarming because it might have an impact on students’ health by increasing the risk of depression, burnout and substance abuse [1, 40]. Consequently the quality of patient care during practical training might be reduced due to decreased empathy and unprofessional behavior [8, 9, 41, 42]. Furthermore, it might reduce academic performance and increase the risk of exiting from medical studies [6, 43]. However, most students in our study still seemed motivated and were looking forward to become physicians, which was mainly due to positive experiences during internships and side jobs and due to support by family and friends.

### Strengths and limitations

Strengths of this study include the rich and comprehensive data, which was collected from 68 medical students in 8 focus groups until thematic saturation was reached. Furthermore, participants of different academic years, age and sex were included to ensure that a broad range of potential opinions and experiences are taken into account. It needs mentioning though that the proportion of female participants was 77%, which is higher than the actual proportion at this particular medical school (i.e. 62 to 63% female students during the period of data collection). Therefore, perspectives of female students may be somewhat overrepresented in our study. Furthermore, the scope of information may be limited by the fact that students of only one university were included. Some themes might therefore relate to conditions specifically linked to our university (e.g. stressors like commuting and organizational factors), whereas others might have been overlooked. However, findings from previous qualitative research and from our study are similar (e.g. stressors like exams and time and performance pressure) and are consistent with established stress models (e.g. effort-reward imbalance model), which may suggest partial transferability of our study results to students at other medical schools. In addition, our study allows for a local view on the study conditions and may thereby help to identify setting-specific intervention needs and means for effective improvement. Furthermore, only students who experienced increased stress may have participated due to higher interest in the research question. Alternatively, it may also be possible that especially students who do not experience major stress have participated due to larger perceived time resources. However, we assume that the likelihood of selection of particularly stressed or non-stressed individuals was minimized by randomly approaching whole course groups to participate in the study.

The fact that participants knew each other, followed the same courses and that a teaching staff member (TM) acted as focus group facilitator may be further limitations of this study. For example, study participants might have felt uncomfortable to raise and share certain topics due to reduced anonymity within focus groups. However, protection and confidentiality of participants’ identity was assured by addressing participants only by numbers instead of names. Furthermore, we experienced that even sensitive topics, such as fear of failure or views about particular medical staff members, were openly discussed. In addition, familiarity within the group ensured that every study participant was actively involved in the discussion. Furthermore, most themes were brought up without any guidance through the facilitator, which reduces the potential influence of the facilitator on study participants.

Due to a high numerus clausus in Germany (i.e. a grade cut-off that governs admission to medical studies), most students accepted for medical studies have graduated from high school with top grades and thereby likely have high confidence in their academic abilities. At medical schools however, a considerable number will for the first time experience average grades or even failure on exams despite high effort. The high confidence in one’s academic skills is likely perceived incompatible with the fact that one failed an exam and may induce an unpleasant state of cognitive dissonance [44]. Attributing one’s failure to external aspects could be one approach to reduce such dissonance. This may explain why the medical students in our study rather discuss organizational aspects of their studies instead of personal characteristics. In addition, students may have preferably come up with suggestions for organizational improvements, because consideration of individual-level interventions may imply that individual deficiencies are discussed, which is socially undesirable, especially when surrounded by other top-grade high school graduates.

Due to considerations regarding time and costs, complete coding was only performed by one person (JW). This approach may be criticized in terms of reliability. However and in line with previous suggestions, the coding scheme and some selected parts of the data material were reviewed by a second researcher (AL) with profound experience in the field of qualitative research [45]. As only minor adaptations were proposed during this review process, multiple coding of the complete data material was deemed unnecessary.

### Implications

Interventions will only be successful when they are perceived to be useful by medical students themselves. We have therefore explored their suggestions for improvement and received a wide range of recommendations. Most of those suggestions highlighted the wish for organizational-level interventions instead of individually focused prevention. Regarding the significant contribution of organizational factors to students’ distress and the fact that most of these organizational stressors seemed modifiable, we propose that organizational structures should be the first target for adaptations. As suggested by some of our study participants, lack of information may easily be reduced by the availability of one single online portal which includes all relevant information as well as information sessions and earlier announcement of exam dates. Furthermore, the presentation of specific learning objectives may be another straightforward approach to reduce students’ uncertainty of what is expected. This is in line with findings from another qualitative study [24]. Furthermore, stronger interweaving of practical and theoretical training as well as preclinical and clinical education may further reduce students’ distress and increase learning effects. Such approaches are already being implemented in new curricula by an increasing number of German medical schools. Insights into the potential positive effects of those new curricula on students’ health are of interest. Also in light of previous qualitative and quantitative research [37–39], reflection on the social climate in medical schools and within the medical society as a whole seems justified. A respectful and supportive climate including better supervision of medical students during practical training might contribute to improved learning environments and subsequently to enhanced patient care in the future.

To develop tailored interventions, separate consideration of students in the old and new curriculum might be of interest due to the possibility that stressors, resources and suggestions for improvement differ between those groups. However, separate analyses were beyond the scope of this report and might be carried out in the future.

Even though our results are in line with previous research findings on this topic, we acknowledge that some of our findings may be less relevant to other universities. Our study shows that focus groups are an efficient research tool to identify stressors, resources and suggestions for improvement in educational contexts. This approach may be useful for other universities to determine specific intervention needs and to thereby reduce students’ distress. At our university, a new project relating to health management for medical students was recently initiated. This project aims to improve study conditions through the development and implementation of support services for students who are in need. In addition, organizational-level (e.g. curriculum, communication) and individual-level (e.g. stress management, relaxation and learning techniques) preventive measures are currently being devised in a participatory approach involving medical students and other relevant stakeholders. The results of this study are thereby a first step to identify promising starting points for interventions.

## Conclusions

In light of evidence documenting medical students’ poor mental health, insights into specific stressors, resources and starting points for interventions are highly relevant. In this qualitative study, previously identified stressors and resources to cope with increased stress during medical studies were confirmed. This implies that these factors are generally important for students at various medical schools in different countries. Furthermore, some new themes emerged such as a burden due to organizational factors and repeat exams. According to students’ perspectives, approaches to reduce students’ distress should predominately involve organizational-level interventions.

## Additional files

Additional file 1: Focus group guide. (PDF 463 kb)

Additional file 2: Completed checklist of the consolidated criteria for reporting qualitative research (COREQ). (PDF 310 kb)

Additional file 3: Additional quotes of participants related to stressors, resources and suggestions for improvement. (PDF 258 kb)

## Data Availability

Full transcripts of focus groups are not publicly available to protect the privacy of our study participants.

## Abbreviations

AL: Adrian Loerbroks
CB: Christin Bergmann
COREQ: Consolidated criteria for reporting qualitative research
ERI: Effort-reward imbalance
FG: Focus group
JW: Jeannette Weber
PA: Peter Angerer
Q: Quote
SS: Stefanie Skodda
TM: Thomas Muth
Y: Year

## Footnotes

1 the balance between private and academic life was beyond the scope of this article and is addressed in a separate report (Bergmann C, Muth T, Loerbroks A. Medical students’ perceptions of stress due to academic studies and its interrelationships with other domains of life: a qualitative study. Med Educ Online. 2019;24 (1):1603526.27)
