# Supplementary material for: Stressors and resources related to academic studies and improvements suggested by medical students: a qualitative study
Source: BMC Med Educ. 2019 Aug 20;19:312. doi: 10.1186/s12909-019-1747-z (PMC6701044; doi:10.1186/s12909-019-1747-z)
Supplement: Supplementary file 1 — Focus group guide. (PDF 463 kb) [file 12909_2019_1747_MOESM1_ESM.pdf]

<u>**Additional File 1**</u>

**Focus group guide**

**1) Introductory question:**

"When you think about your medical studies, how does that make you feel? …
I would like to learn how you experience your studies on a day-to-day basis. What had been challenging? What had been helpful? …."

"What comes to your mind when you think about stress and academic studies?"

<u>Note to facilitator: enable everyone to speak, but not necessarily in a fixed order. Ensure that discussions do not wander off to a general discussion of study conditions or evaluation of specific courses</u>

**2) Key questions regarding stress and coping:**

a) When you think about the last time you have felt under pressure due to your studies, what happened that day?
b) How did that happen?
c) How did you deal with it?
d) What are your suggestions to avoid or reduce stress related to your academic study? What would you like to change?
   I. Regarding study conditions
   II. Regarding medical students
e) Do you also experience positive aspects related to your medical studies? What are these positive aspects?

**3) Key questions regarding stress and private life**

a) How do you experience the combination of academic life and private life?
b) Is there any conflict between the two?
c) What type(s) of conflict(s)?
d) How do they come about?
e) How did you deal with it?

<u>*Note to facilitator: please ensure that both directions of conflict are discussed, i.e. studies → private life and private life → studies*</u>

**4) Outlook**

f) How do you view the topics "work stress" and "work stress versus private life" when you envision your future career, e.g. working as a medical doctor?

**5) Summary**

g) What do you personally regard as most important related to stress due to academic studies and its interrelatedness with one's private life? / What are your take home messages for us?

**6) End**

h) Is there anything else that is important for you and has not been discussed so far?
i) Thank you for this good discussion
