# Supplementary material for: Stressors and resources related to academic studies and improvements suggested by medical students: a qualitative study
Source: BMC Med Educ. 2019 Aug 20;19:312. doi: 10.1186/s12909-019-1747-z (PMC6701044; doi:10.1186/s12909-019-1747-z)
Supplement: Supplementary file 2 — Completed checklist of the consolidated criteria for reporting qualitative research (COREQ). (PDF 310 kb) [file 12909_2019_1747_MOESM2_ESM.pdf]

Additional File 2

Completed checklist of the consolidated criteria for reporting qualitative research (COREQ; (1))

No	Item	Description
**Domain 1: Research team and reflexivity**		
Personal characteristics		
1.	Facilitator	Thomas Muth
2.	Credentials	Jeannette Weber (MPH), Stefanie Skodda, Dr. Thomas Muth, Prof. Dr. Peter Angerer, PD Dr. Adrian Loerbroks
3.	Occupation	JW: research associate; SS: medical student; TM, AL: senior research associate; PA: university professor
4.	Gender	JW, SS: female; TM, PA, AL: male
5.	Experience and training	JW: educational background in biomedical science and public health, practical experience in occupational health research; SS: medical student; TM: educational background in psychology and public health, experience in occupational health research, qualitative research and teaching; PA: educational background in human medical studies, experience as a clinical doctor and in occupational health research; AL: educational background in epidemiology and health sciences, experience in occupational health research, qualitative research and teaching
Relationship with participants		
6.	Relationship established	No
7.	Participant knowledge of facilitator	Participants knew that TM was working as a research associate on student health
8.	Facilitator characteristics	No other characteristics were reported about the facilitator
**Domain 2: Study design**		
Theoretical framework		
9.	Methodological orientation and theory	Qualitative content analysis by Mayring
Participant selection		
10.	Sampling	Convenience sampling
11.	Method of approach	Via social media or personal contact
12.	Sample size	68 participants
13.	Non-participation	Not applicable
Setting		
14.	Setting of data collection	Conference room at university
15.	Presence of non-participants	Two doctoral students who took field notes
16.	Description of sample	$Mean_{age}$=24 (range: 18-34 years); female = 77%, male = 23%
Data collection		
17.	Interview guide	Provided as supplemental material
18.	Repeat interviews	None
19.	Audio/visual recording	Audio recording
20.	Field notes	Yes
21.	Duration	Circa 90 minutes
22.	Data saturation	Yes
23.	Transcripts returned	No
**Domain 3: Analysis and findings**		
24.	Number of data coders	Two
25.	Description of coding tree	Provided as supplemental material
26.	Derivation of themes	Deductive coding: Stressors, resources, suggestions for improvement; all other categories were inductively coded
27.	Software	MaxQDA 12
28.	Participant checking	No
Reporting		
29.	Quotations presented	Yes
30.	Data and findings consistent	Yes
31.	Clarity of major themes	Yes
32.	Clarity of minor themes	Yes

1. Tong A, Sainsbury P, Craig J. Consolidated criteria for reporting qualitative research (COREQ): a 32-item checklist for interviews and focus groups. International Journal for Quality in Health Care. 2007;19(6):349-357.
