# Supplementary material for: Stressors and resources related to academic studies and improvements suggested by medical students: a qualitative study
Source: BMC Med Educ. 2019 Aug 20;19:312. doi: 10.1186/s12909-019-1747-z (PMC6701044; doi:10.1186/s12909-019-1747-z)
Supplement: Supplementary file 3 — Additional quotes of participants related to stressors, resources and suggestions for improvement. (PDF 258 kb) [file 12909_2019_1747_MOESM3_ESM.pdf]

Additional File 3

Quotes of the participants related to stressors, resources and suggestions for improvement

Stressors

	Focus group	Study year	Quote
Q1	FG 4	1	I have similar experiences. I also commute. It takes me three hours every day. An hour and a half to go there and another hour and a half to go back. And when I come to attend lectures for an hour and a half, then I always feel that I don't get anything done in the morning and I don't get anything done in the evening. I think we are involved with a very rigid system here where everything is set. The schedule is set. We can't choose anything freely.
Q2	FG 1	5	And then having separate written exams, to be honest that drives me crazy, because you collect your credits but you don't know exactly what to prepare for and then you only have one more written exam and you still don't know if you'll get the admission for the final exam.
Q3	FG 4	1	There is a kind of balance with I'm going to quit. It's too much for me (other participants: Yes) and this existential fear, that one really (.) I'm afraid that I will not pass this exam, because then my stress will increase even more, because then I will have to prepare for it again and also start learning for the new one and if I fail the new one, then I will have to do two more exams and it becomes increasingly difficult. I am really very scared that I will not pass the written exams.
Q4	FG 2	5	I think we also get our share of the fact that the general work conditions in the hospitals are (,) well I (,) on every second ward people complain that staff was reduced, we don't have enough employees, and that they can't supervise us is something I can explain well to myself. I think we are not a priority for most of them (other participants: approval), and I can somehow understand this, I just think we get our share, and that's particularly stupid for us.
Q5	FG 5	2	I think teaching in this module is incredibly bad. Well, it simply is. There is not a single subject where I can say, OK, I feel well-prepared.
Q6	FG 4	1	For example, and this was very bad during the last module, I sat there completely apathetically and I thought I hate this course because intellectually (!) Intellectually, I feel I am not challenged enough while at the same time there is an enormous amount of knowledge I need to absorb and I'm not in the mood for doing it because it's not fun and it isn't interesting. It's not as if you could (!) As if you would write an essay now and collect all kinds of facts and connect them here and there and oh, how nice. That's how it is (knocks on table).
Q7	FG 1	5	Yes, everyone always tells me that they want to start early, but no one begins studying on the first day and in the end, you have about two weeks for studying for the exam, for subjects that are really big and important, but somehow there's a lack of time.
Q8	FG 8	2	I try, I do try to understand everything, because I want to understand it, but from a certain point on that doesn't work the way I do it and then I'm a bit disappointed, because somehow when you (,) when you study you try to, because you look for more information and you try to find ways to be informed, but there's no time for doing this.
Q9	FG 8	2	It's like this, when it becomes too much, then you are not motivated anymore, because then (,) if you had 30% more time, then you could study peacefully and without stress, that would surely be great. But as it is you feel exhausted and worn out and you really don't want to go on anymore.
Q10	FG 5	2	But all in all, at the moment I think the atmosphere is really bad. During all of the semester. I find the atmosphere is really bad. I almost don't want to attend anymore because I wonder what's going to happen today or who's going to freak out next. Well, I find the atmosphere so bad that I don't have

			fun anymore. Well, I study because I wanted to and I was looking forward to it, learning it all and so on. But in the meantime, I find co-operation, that's something that isn't really there anymore, people are destroying everything for each other. Sometimes, there's just jealousy. People begrudge each other things. Or they feel attacked and at the moment I think this is really terrible.
Q11	FG 2	5	I think some of the stress of studying is caused by what one expects from oneself.
Q12	FG 7	5	So many things come together. You think it's expensive because of the fees. When you fail you can't do the next semester. Then your friends will be gone and you will have to study the same topics again. In addition, you're basically good or have mostly been successful and then you're afraid that you might fail. And if you failed once, it could happen again. Really, if you failed once, you always know in the back of your mind that you only have two more chances and if they don't work out you will have to quit.
Q13	FG 1	5	The problem is that it takes so long. I don't know about you, but I have a student loan and that creates a certain financial pressure to finish within a certain amount of time because one (other participant: At some point in time BaFög [German federal financial aid for students] will decline). Because from a certain point on you will not receive any support or another loan anymore.
Q14	FG 3	5	And then I also did (,) for whichever reason, accept a doctoral thesis and I am completely overburdened.

Resources

	Focus group	Study year	Quote
Q15	FG 1	5	Well, basically what I find good is that there are seminars for which I can decide if I want to attend or not (,) if I go there and I don't find it interesting, I think there's no point in attending, then I won't attend anymore. It's the same with lectures. I think there's no point in lectures, at the age of twenty or twenty-five it's my decision to say, there's no point in it, I won't attend, I will study this at home alone.
Q16	FG 7	5	Also, that the pressure was gone. That you knew you can fail and then repeat it in the last module. Just knowing this helps so much.
Q17	FG 7	5	During the first two semesters, I did clinical electives except for a week or two. Firstly, I loved it. This was the first time I thought, wow, that's close to practicing and I was allowed to do much more and it worked out. And then you realized that you get better and your skills improve and things like that. And I could easily apply all kinds of things and I also found it less troubling.
Q18	FG 8	2	Well, at the moment the preliminary physical examination is a ray of hope and afterwards it will get better (group laughs).
Q19	FG 8	2	When worse comes to worse, sure, everyone feels low sometimes, then I always call my parents, or I just pack my things and go home for a day, because it's such a great support.
Q20	FG 3	5	In any case it's people you talk to who passed successfully because then you see that it's possible, uh, you can survive it.
Q21	FG 3	5	I think fellow students are most helpful with this, if there's someone who tells you, look, you have to register for the exam here or you can cancel there.
Q22	FG 2	5	This is my basic attitude, I dealt with it early on. It's a kind of subject (,) we study this to be able to help people later on and I don't have to make myself ill because of it and, uh, if it's not possible, then it's not possible, then that's how it is.
Q23	FG 5	2	Personally, I dealt with exams and studying in a more relaxed manner. And I think so far it has always worked out. It's going to be alright.
Q24	FG 1	5	And we knew that what we do during the internship is something that the lecturers take very seriously and I think that makes you take it seriously as well and you can handle the subject matter better, not taking into account the amount of subject matter, but you can process it better.

Q25	FG 4	1	I like the preliminary test because for people who always procrastinate, they need to read the lecture notes and to deal with them in order to pass the test and that's a good thing. That helps to structure the whole thing.

Suggestions for improvement

	Focus group	Study year	Quote
Q26	FG 4	1	That you have the freedom of choice. Also with regard to the schedule, so that for example a seminar, I don't know. Tuesday afternoon. On Wednesday, there will be another one. That you can choose from the schedule. This one I would like to attend then. I would like to attend that one then but we always decided this as a group. We decided on a specific point in time and then we have to attend. […] This somehow keeps me from really dealing with it. Then I studied anatomy in the morning. I go to a seminar on a totally different subject and that has nothing to do with anatomy, instead of attending the seminar when I chose to do it and when I have time to prepare.
Q27	FG 1	5	Well, it's good that there are lectures because anyone can decide on whether to attend or not, but it's (,) or also seminars, it's good that this exists, the option to, because I think that the fact that the university offers this, that's how it has to be, teaching is their mission, but I don't think you should force everyone to attend.
Q28	FG 7	5	We really have a lot of free time, even without taking time off, but sure, you look forward to the semester break. You think, wow, cool, I won't have to enter this building for three months and so on. But on the other hand, it's a disadvantage that there are so many obligations. For three or two months, I have to squeeze everything in this period of time. Of course, we have to do internships, but we don't have to study so much theory and do written exams. For example, I find this somehow, of course it is cool to have so much free time but due to it everything takes even longer than necessary. If the structure was less compact one could study many subjects in a better way and then the written exams wouldn't be so difficult and the stress wouldn't be such a burden because when we study for 10, 12 hours then it becomes more and more difficult.
Q29	FG 4	1	I think while studying, that above all these are such demanding and draining studies. You really need time and during the semester break or when there are no lectures there shouldn't be all kind of internships or seminars. I would have preferred it if the seminar on sociology were included with the lecture period instead of scheduling it for the semester break.
Q30	FG 7	5	One approach could be to reduce the group size. Last semester there actually were 15 students in a group. When push came to shove and we did bedside teaching and the group couldn't split, then there were 15 people in a patient's room standing in a circle around the bed and of course nobody places himself next to the patient and says OK, I'll do it, when there are so many people there. It really impacts quality. Even if the lecturer took great pains in preparing everything.
Q31	FG 2	5	I often think what I wish for is just being taken along in daily business, because that's what I want to see, and I don't think that would take too much time.
Q32	FG 8	2	One could create a platform where you can check exams, results, and schedules and anything else.
Q33	FG 6	1	I think exams and tests are enough. It is all part of the final exam of the module that includes all subjects, you don't need any small things in between that just serve for wasting time more or less.
Q34	FG 7	5	What is desirable is to really get basic knowledge of all the different subjects so that one can be a good physician afterwards, even if one specializes in another subject, that one has an overview over the other subjects.
Q35	FG 7	5	What I also. In any case I would like to strengthen all other skills. Well, we (,) well everything we do, we develop skills in a subject, but anything else,

			there’s a lack in everything. For example, practical skills, that’s what you gain from clinical electives. You don’t learn inserting a chest tube by doing it once on a model or introducing a peripheral venous catheter by doing it on each other once. That’s not how you learn it. You merely know that it exists. And so many other skills that I think are not strengthened during our studies like behaviour or self-reliance. I think that within our studies there’s not much space for developing one’s personality, for advancing just for oneself.
Q36	FG 1	5	I also think there are different programmes, but you don’t really know and I would really find it good (,) well I currently attend the module on borderline situations and to be honest you see some unpleasant things there and I think you are left a bit alone with it and I have been studying for ten semesters now and it was only last week that I learned that theoretically there is a contact person for us. You can contact this person if there’s something you can’t deal with well. To be quite honest that’s a bit late. And I think it would be good if there was access to someone I could contact when I (unintelligible) have too much stress, or also when, something that is very helpful in medical studies, when I have a very serious case that affects me mentally, when I participated in an autopsy and didn’t like how the body was disembowelled.
